# Pennsylvania Hospital

**Published:** 1839-02-23

**Authors:** 


### CLINICAL LECTURES.

### PENNSYLVANIA HOSPITAL.

#### AFFECTIONS OF THE LACRYMAL DUCT.

*Saturday, February* 9th.—Dr. Harris introduced a patient affected with an obstruction of the lacrymal duct. Before proceeding to operate, he offered the following remarks:

It was at one time the practice, to call all affections of the lacrymal passages, fistula lacrymalis. There are various diseases located in the inner corner of the eye, distinct in character, and requiring different treatment.

Inflammation is often located in the cellular membrane, covering the lacrymal sac, while the sac itself is free from disease. It sometimes exhibits an erysipelatous form, and terminates in an unhealthy suppuration. In the progress of this disease, the sac may become secondarily affected.

In the treatment of this cellular inflammation, it is particularly important to produce resolution. General and local bleeding, with warm fomentations and aperients, are found most efficacious. When pus has formed, the abscess ought to be early opened, otherwise the lacrymal passages may become involved. When the sac does become so affected, then the treatment is the same as is found necessary in true fistula lacrymalis.

When the lacrymal sac is primarily inflamed, it enlarges, is circumscribed, and painful, more particularly if pressed. The tumour thus formed in the inner canthus of the eye is of a pale red, but, by degrees, assumes a darker colour, and is accompanied with a pain that often shoots into the orbit. The inflammation, if not arrested by appropriate means, extends to the ducts and puncta, thus obstructing them, and causing the tears to flow freely over the cheek.

When these symptoms have continued for a few days, the inflamed mucous lining of the sac secretes an abundant puriform matter. Pressure with the finger on the sac, will commonly cause the pus to flow through the puncta. When the ducts are entirely obstructed, then the swelling increases, its redness assumes a darker hue, and a yellowish, soft spot is seen, which indicates the approach of matter to the surface.

It is now the duty of the surgeon to lay open the abscess, in order to prevent fistulous openings. It is always most prudent to make an artificial opening, rather than to allow the abscess to burst spontaneously, as the matter burrows under the skin, and discharges through an aperture so narrow, that the tears alone escape, while the thicker contents remain behind. It is this condition of the parts, that constitutes true *fistula lacrymalis.*

It is not common that the opening in the integuments corresponds with the ulcerated point in the sac. On the contrary, we have generally a long sinuous passage, extending between the sac and orbicularis muscle. The puriform discharge flows through the orifices, becomes limpid, rather than thick, and whitish. In some few instances the ducts re-open—the tears and mucous are transmitted through the nares, the fistulous openings heal, and a spontaneous cure is effected.

Fistula lacrymalis is sometimes a symptom of disease of the bones in the vicinity of the lacrymal passages. I have a case of this kind, now under my care, which has supervened from the injudicious use of mercury.

In the treatment of suppuration of the sac with epiphora, it is important to attend to the general health. Frequently the habit of the patient will be found weak and strumous. It is the case in the patient on whom I propose to operate this morning. You will observe that he is of a phlegmatic temperament, fair skin, tumid lips, and sandy hair. He had, when brought into this institution, poropthalmia, and diseased digestive organs. By a course of alterative treatment, his general health has improved, the disease of his eyelids has disappeared, and the suppuration of the sac has much diminished. The only local remedies used, have been the application of leeches, washing out the sac with astringent lotions, by means of Anel's syringe, and touching with diluted citrine ointment, the diseased margins of the eyelids. Such means will often cure the disease, and open the obstructed passages. They have not succeeded in this instance, and therefore an operation has become necessary. I have not used Anel's probe with the view of dilating the strictured duct, because I have little confidence in its utility. Though you may often pass the probe through the duct into the nose, it never sufficiently dilates the stricture, so as to produce a permanent cure. You might as well attempt to cure a stricture of the urethra with a pocket probe. No stricture can be properly cured, unless the tube is dilated fully to its natural dimensions. If, then, a cure cannot be accomplished by such means, it is better to open the sac early, and thus prevent more serious mischief.

In performing this operation, I use a straight and narrow bistoury, with a groove on its side. You feel for the firm ligament which attaches the orbicularis palpebrarum to the nasal process of the superior maxillary bone, just below which is located the upper orifice of the ductus ad nasem, and within the edge of the orbit. On this point, the knife is made to pass into the sac, pushed downwards, backwards, and a little forwards, in the direction of the duct. A probe should be now passed along the groove in the bistoury, until it is fairly lodged in the duct. Force is sometimes required to overcome the stricture, but if made in a proper direction, so as not to injure the covering membrane of the bone, or the bone itself, so harm can result.

When the operation is completed, a small quantity of blood and matter will issue from the corresponding nostril.

In order to secure a pervious state of the nasal duct, various methods have been proposed. Of the inefficacy of Anel's probe passed through the puncta, I have already spoken. When thick flocculent matter, secreted by the sac, obstructs the duct, syringing the passages from the puncta has completely removed the disease. I have seen many examples of this kind. Inserting a curved probe under the turbinated bone, will often remove trifling obstructions, particularly if followed by injections through the same channel. This, however, is an operation requiring much tact, and which ought not to be attempted until the operator has repeatedly practised on the dead subject. The first attempt to introduce the instrument is always the most difficult, as the valvular projection of the membrane at the lower orifice forms a considerable obstruction. So soon as this valve is destroyed, subsequent introductions become more easy.

The preferable plan of treating this disease, is by opening the sac in the manner already indicated, and afterwards to use either tubes or styles. The tubes are constructed of either silver or gold, and of equal calibre throughout their whole extent. Notwithstanding the high recommendation of this instrument by Decypreter, its effects are often unsatisfactory and annoying. The tube frequently becomes obstructed by the concretion of the matter passing through it. To remove this obstruction is by no means easy. A free incision over the tube is rendered necessary, and, with a strong forceps, the instrument must be seized and drawn out, which is commonly a very painful operation. There are few surgeons who have had much experience with this instrument, who are not disposed to abandon its employment.

After some experience, I vastly prefer the style to the tube. They may be made of either gold, silver, pewter, or lead. After a probe is passed through the duct into the nose, it should be withdrawn, and a small style introduced in its place. The size should be gradually increased until it is of sufficient magnitude to correspond with the calibre of the duct in its natural and healthy condition.

The plan on which I propose to operate in this case is, to dilate the stricture with a silver probe, and after withdrawing it, to substitute a catgut bougie, and allow it to remain several days. The advantage of this instrument is, that by the absorption of moisture, it becomes flexible, unirritating, and increases to double the size of it when first introduced. In this manner you fully dilate the duct, and afterwards introduce the leaden style, which produces less irritation than other metals. This leaden instrument should be worn until all irritation and suppuration entirely ceases. At this time, the style may be removed, the aperture in the integuments and over the sac closed, and thus will ensue the permeability of the canal.

[The patient now submitted to the operation already proposed.]

